# Supplementary material for: Causal Relationship between Aging and Anorexia Nervosa: A White-Matter-Microstructure-Mediated Mendelian Randomization Analysis
Source: Biomedicines. 2024 Aug 16;12(8):1874. doi: 10.3390/biomedicines12081874 (PMC11351342; doi:10.3390/biomedicines12081874)
Supplement: Supplementary file 1 [file biomedicines-12-01874-s001.zip › Supplementary Figure/Figure S9/Leaveoneout plot of MR analysis for ubm-b-1954 to AN.pdf]

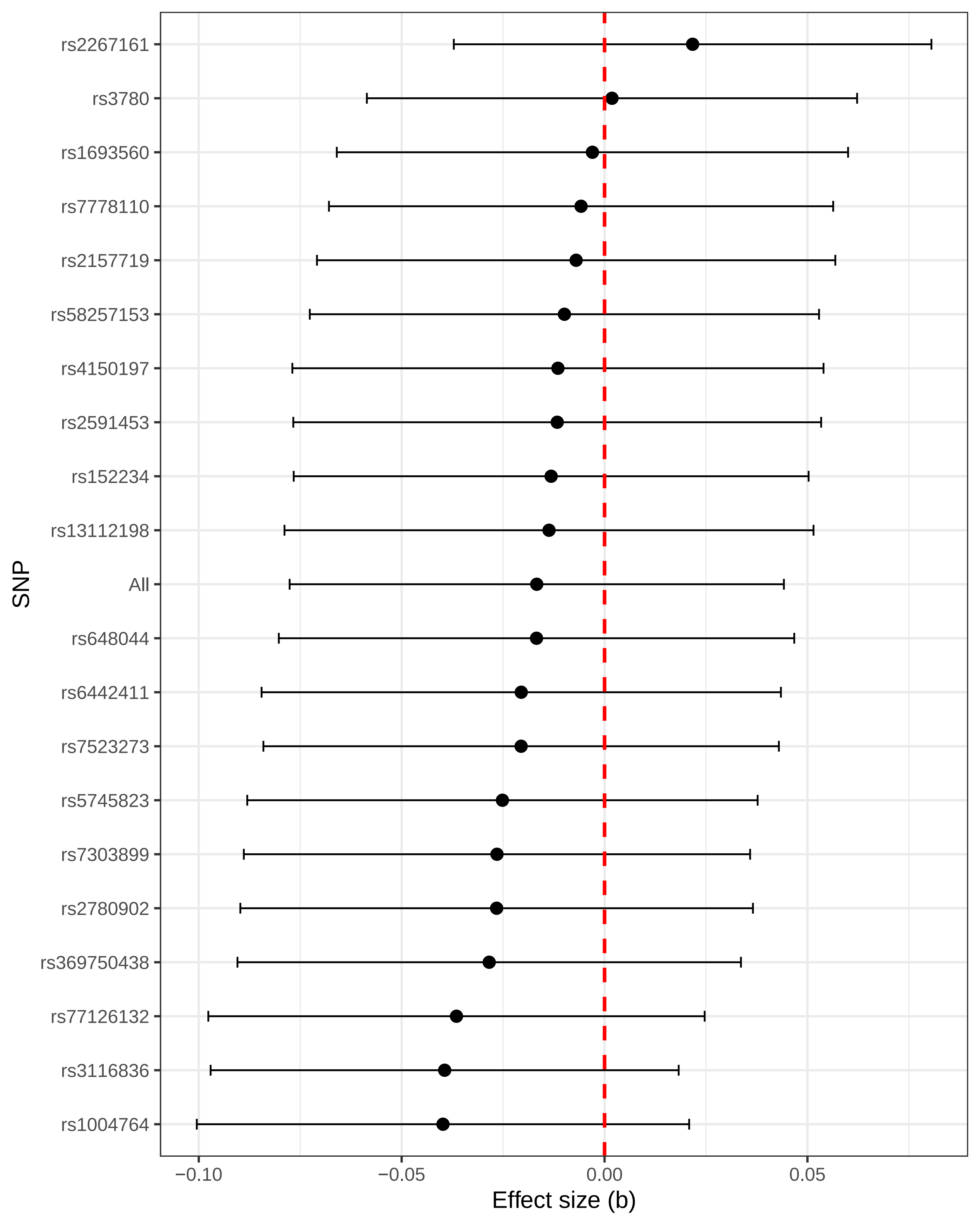
rs2267161
rs3780
rs1693560
rs7778110
rs2157719
rs58257153
rs4150197
rs2591453
rs152234
rs13112198
All
rs648044
rs6442411
rs7523273
rs5745823
rs7303899
rs2780902
rs369750438
rs77126132
rs3116836
rs1004764
SNP
−0.10
−0.05
0.00
0.05
Effect size (b)
